# Macrophage Infiltration Reduces Neurodegeneration and Improves Stroke Recovery after Delayed Recanalization in Rats

**DOI:** 10.1155/2022/6422202

**Published:** 2022-08-17

**Authors:** Jinwei Pang, Nathanael Matei, Jianhua Peng, Wen Zheng, Jing Yu, Xu Luo, Richard Camara, Ligang Chen, Jiping Tang, John H. Zhang, Yong Jiang

**Affiliations:** ^1^Department of Neurosurgery, The Affiliated Hospital of Southwest Medical University, Luzhou, Sichuan 646000, China; ^2^Sichuan Clinical Research Center for Neurosurgery, The Affiliated Hospital of Southwest Medical University, Luzhou, Sichuan 646000, China; ^3^Department of Anesthesiology, Neurosurgery and Neurology, School of Medicine, Loma Linda University, Loma Linda, CA 92354, USA; ^4^Luzhou Key Laboratory of Neurological Diseases and Brain Function, The Affiliated Hospital of Southwest Medical University, Luzhou, Sichuan 646000, China

## Abstract

**Background:**

Recent cerebrovascular recanalization therapy clinical trials have validated delayed recanalization in patients outside of the conventional window. However, a paucity of information on the pathophysiology of delayed recanalization and favorable outcomes remains. Since macrophages are extensively studied in tissue repair, we anticipate that they may play a critical role in delayed recanalization after ischemic stroke.

**Methods:**

In adult male Sprague-Dawley rats, two ischemic stroke groups were used: permanent middle cerebral artery occlusion (pMCAO) and delayed recanalization at 3 days following middle cerebral artery occlusion (rMCAO). To evaluate outcome, brain morphology, neurological function, macrophage infiltration, angiogenesis, and neurodegeneration were reported. Confirming the role of macrophages, after their depletion, we assessed angiogenesis and neurodegeneration after delayed recanalization.

**Results:**

No significant difference was observed in the rate of hemorrhage or animal mortality among pMCAO and rMCAO groups. Delayed recanalization increased angiogenesis, reduced infarct volumes and neurodegeneration, and improved neurological outcomes compared to nonrecanalized groups. In rMCAO groups, macrophage infiltration contributed to increased angiogenesis, which was characterized by increased vascular endothelial growth factor A and platelet-derived growth factor B. Confirming these links, macrophage depletion reduced angiogenesis, inflammation, neuronal survival in the peri-infarct region, and favorable outcome following delayed recanalization.

**Conclusion:**

If properly selected, delayed recanalization at day 3 postinfarct can significantly improve the neurological outcome after ischemic stroke. The sanguineous exposure of the infarct/peri-infarct to macrophages was essential for favorable outcomes after delayed recanalization at 3 days following ischemic stroke.

## 1. Introduction

Acute ischemic stroke (AIS) is a global health concern that often leads to lifelong disability or death of patients [1, 2]. Although vascular recanalization therapy is widely recognized to be effective for AIS patients with large vessel occlusions (LVO), it is limited in the clinical setting due to the required window of time between ischemia onset and intervention [1, 3–5]. Previously, AIS reperfusion therapy has been restricted to a 4.5 h window for rt-PA thrombolysis or 6 h for endovascular thrombectomy [6]. Despite these limitations, early arterial recanalization following AIS has been shown to improve cerebral blood perfusion, functional outcome, and survival. The conventional window for reperfusion has not been challenged beyond these time points mostly due to the risk of ischemia/reperfusion injury [7], hemorrhagic transformation risk [8], early arterial reocclusion [9], and other associated deleterious outcomes [10]. Most AIS patients fail to receive intervention due to delays that increase the door-to-needle time (DNT) or door-to-puncture time (DPT), e.g., failure in early diagnosis, onset of unidentified symptoms, and/or other associated limitations in current stroke protocols [11, 12]. The majority of AIS patients presenting with LVO do not receive recanalization therapy and experience unfavorable disability and mortality. New therapeutic strategies are thus urgently needed for AIS.

Following advances in brain imaging to identify salvageable tissue, thrombolysis therapies and endovascular thrombectomy have shown remarkable improvement in outcomes following delayed recanalization beyond 6 h in AIS patients with LVO [13]. Two landmark randomized controlled clinical trials (DAWN and DEFUSE 3) reported that selective delayed recanalization up to 24 hours after symptom onset via endovascular mechanical thrombectomy resulted in favorable outcomes [14, 15]. Moreover, endovascular thrombectomy for AIS patients beyond 24 hours from symptom onset has also been shown to be safe and effective [16]. In the EXTEND trial, evidence has shown thrombolysis with rt-PA to be effective and improve overall outcome when administered in a window of 4.5-9 hours after symptom onset [17, 18]. Along these lines, imaging-based intravenous thrombolysis with Tenecteplase is efficacious up to 24 hours after symptom onset in selected patients [19]. A study reanalyzed the data of the DEFUSE 3 trial and suggested that approximately 20% of patients with LVO who are not treated with thrombectomy had a salvageable penumbra for at least an additional 24 hours [20].

Notably, the long-term outcomes of patients who received recanalization beyond 6 hours were reported to be improved as well [16]. In a follow-up study of the REVASCAT trial, the difference in outcomes of patients in the endovascular therapy group versus standard therapy group continued to increase from 3 months to 1 year [21]. As most patients are assessed for possible treatment up to 24 hours from symptom onset, two questions remain: (1) can more accurate criteria be established to categorize patients evaluated within 24 h from symptom onset for possible treatment; (2) would patients with salvageable brain tissue beyond the 24 h window also benefit from “delayed recanalization”?

Basic science research has reported that delayed recanalization at 3 days after permanent middle cerebral artery occlusion (pMCAO) attenuated neuronal apoptosis and improved functional outcome in rats [22]. Paralleling these studies, others have reported that even recanalization at up to 14 days after middle cerebral artery occlusion improved rat sensorimotor function although no difference in infarct volume was observed compared to controls [23]. Taken together, delayed recanalization can potentially limit cell death and reduce neurological deficits associated with ischemia. Nevertheless, the cellular and molecular mechanisms surrounding delayed recanalization have not been fully characterized.

Following delayed recanalization and although not fully confirmed, many peripheral circulation factors and/or cells are likely necessary for vascular remodeling and cerebral repair of the infarct core and perilesion regions [24]. In support, peripheral circulation macrophage infiltration reduces unfavorable outcomes in transient MCAO models [25, 26]. Furthermore, evidence supports that sanguineous exposure to macrophages may promote tissue repair in ischemic tissue via accelerated angiogenesis [26, 27]. However, the role of peripheral circulation macrophages on delayed brain repair has not been evaluated. In this study, we aim to elucidate whether the underlying benefits of delayed recanalization on neurological recovery are mediated through macrophage-dependent angiogenesis in a rat MCAO model.

## 2. Methods

### 2.1. Laboratory Animals

Adult male Sprague-Dawley rats (260–280 g) were housed and treated in a humidity and temperature-controlled environment with regular light/dark cycle and free access to food and water. All animal procedures were approved by the Loma Linda University and the Southwest Medical University Institutional Animal Care and Use Committee (IACUC) and in accordance with Stroke Treatment Academic Industry Roundtable (STAIR) guidelines and the National Institutes of Health Guide for the Care and Use of Laboratory Animals.

### 2.2. Experiment Design, Excluding and Including

The current study consists of two parts. Based on previous studies [22], we performed recanalization 3 days after MCAO. Detailed group information and a schematic illustration of the experimental process are displayed in additional file 1: Fig. S1. Sample sizes were calculated according to a previously reported [28] (formula: 10 ≤ *n* × *M* − *M* ≤ 20 (*n* means the number of animals in each group, and *M* means the number of groups)). A power of 0.8 and *α* of 0.05 were used to choose the sample size.

First, rats at 3 d after MCAO were allocated randomly into either pMCAO or rMCAO groups. However, we found that some animals with poor neurological function could not tolerate recanalization surgery with resultant unfavorable outcomes, including death. MRI scanning revealed that poor neurological performance may be related to hematoma volume expansion. Therefore, animals with neurological scores less than 6 at day 3 after MCAO were excluded from the study. The remaining rats were allocated randomly into pMCAO or rMCAO groups. Detailed information about experimental and procedural design is listed in supplementary material (Additional file 1: Table S1).

### 2.3. Establishment of Middle Cerebral Artery Occlusion (MCAO) Model and Recanalization

Cerebral blood flow (CBF) monitoring was used to ensure MCAO model alignment with previous methods [23]. Briefly, rats were anesthetized with an intraperitoneal injection of ketamine/xylazine mixture (80/20 mg/kg) and subcutaneous injection of atropine (10 mg/kg). A scalp incision was made, and a burr hole was created over the MCA territory. CBF was measured via a laser Doppler flow probe (OxyFlo probe, MNP100XP, AdInstruments Inc., Colorado Springs, CO, USA) to obtain the baseline and post-MCAO CBF levels using specialized software (PowerLab PL3504 and LabChart Pro, AdInstruments Inc., Colorado Springs, CO, USA). After acquiring the CBF baseline, the MCAO surgery was initiated. An abrupt attenuation of CBF was the mark of successful vessel occlusion. CBF was continuously monitored during the MCAO surgery and recanalization processes.

All MCAO model surgical procedures were performed as previously described [23]. In permanent MCAO (pMCAO) groups, an incision in the neck was made, and the right common carotid artery (CCA), external carotid artery (ECA), and internal carotid artery (ICA) were exposed. Next, a 4-0 monofilament suture with a silicon-coated tip was inserted into the middle cerebral artery (MCA) bifurcation from the ECA stump. The occlusion suture was permanently tied, and the excess thread was cut off. Next, the neck wound and scalp incision were sutured. All rats were separately placed into 37°C recovery chambers for anesthesia recovery. In delayed recanalization animal (rMCAO) groups, the scalp incision and neck wounds were reopened, and the occlusion suture was removed at 3 d following MCAO. Sham animals underwent all surgical procedures without occlusion.

### 2.4. Brain Edema and Hemorrhage Risk Evaluation

Brain edema was evaluated by magnetic resonance imaging (MRI), and brain water content was measured as previously reported [29]. Details for MRI scanning have been described in the following section. Brain water content was measured by the dry/wet method. Briefly, rats were killed 3 or 4 days after MCAO surgery and 1 day after recanalization. Left and right hemisphere and cerebellum brain samples were collected. Each specimen was weighed to obtain the wet weight and then weighed again after drying in an oven at 105°C for 24 h to obtain the dry weight. Brain water content (%) was calculated as [(wet weight − dry weight)/wet weight] × 100%. Hematoma was identified as hypointense (susceptibility) signal in brain parenchyma on MRI-T2 imaging. Hematoma incidence was estimated using incidence on MR imaging while hematoma expansion was defined by calculating the hematoma volume change outlined on MRI-T2 images.

### 2.5. Magnetic Resonance Imaging (MRI) and PET-CT Scanning

MRI and PET-CT were performed as previously described [29, 30]. MRI with T2-weighted imaging (T2WI) and diffusion weighted imaging (DWI) enable sensitive, reliable detection of hematoma and brain edema volume, respectively. To evaluate the effects of delayed recanalization on brain edema and hemorrhagic risk outside of the conventional time window after AIS, we performed MRI with T2WI and DWI using a Bruker 7.0 T system (Bruker Biospin, Billerica, MA, USA). T2-weighted images were acquired using RARE (repetition time = 4000; echo time = 45; RARE factor 8, 1 mm; field of view = 40 mm × 40 mm; and an acquisition matrix size of 256 × 256). Diffusion-weighted images (DWI, TR/TE = 2500/22 ms) were obtained using a 2D spin echo-planar imaging sequence (SE-EPI) with a FOV of 40 × 40 mm and an acquisition matrix size of 128 × 128. Apparent diffusion coefficient (ADC) maps were calculated based on three different *b* values (0 s/mm^2^, 500 s/mm^2^, and 1000 s/mm^2^). T2 and DWI-hyperintensity volumes were analyzed using the Bruker Paravision 6.0 software by a blinded investigator.

To assess reperfusion and determine the influence of delayed recanalization on cerebral glucose uptake after AIS, a preclinical micro-PET-CT scanner (Inveon MM gantry, Siemens, Germany) was used to measure cerebral glucose uptake at 3 days post-AIS, just before recanalization and 24 h after recanalization, as previously described [30]. The cerebral glucose uptake ratio was evaluated by standardized uptake value (SUV). SUV = Ct/ID∗Wt.Ct (MBq/cm^3^) represents the decay-corrected activity concentration of the tested brain regions. ID (mCi) represents the injected dose of ^18^F-FDG, and Wt (kg) indicates the weight of the rat.

### 2.6. Neurobehavioral Tests

Sensorimotor and cognitive function were measured by beam walking balance, modified neurological scale score, and Morris water maze test by a blinded investigator as previously described [23]. The neurological scale score consists of 6 parts: spontaneous activity, symmetry in the movement of four limbs, forepaw outstretching, climbing, body proprioception, and response to vibrissae touch with a maximum score of 18. Beam walking balance test score is based on walking performance on a suspended balance beam. Maximum score is 5 with a higher score indicating better neurological function.

For cognitive function evaluation, the Morris water maze test was performed at 1 month after MCAO establishment or delayed recanalization as previously reported [31]. Briefly, each rat performed 5 trials each day from 1 d to 5 d (blocks 1-5). Each rat was well trained and guided to find the platform from 1 d to 5 d within 1 minute in the cued and spatial tests. Escape latency and path length were recorded from blocks 1 to 5. In the probe trial (day 6), the platform was removed, and 60 seconds was allotted to measure the percentage of time spent in the platform-quadrant. Computerized tracking system was used to record the number of platform crossings (Noldus Ethovision; Noldus, Tacoma, WA, USA).

### 2.7. Assessment of Cerebral Infarction Volume

Cerebral infarction volume was measured as previously reported [31]. The area of each slice was calculated using the following formula: (area of contralateral − area of noninfarcted ipsilateral tissue)/2 × area of contralateral × 100%. Infarct area was calculated for each slice, and to determine the percentage of the whole brain infarct volume, the average of all slices was taken to represent the percentage of the infarcted volume for each animal [32, 33]. Briefly, animals were perfused with ice-cold PBS. Brains were then removed, and six 2 mm thick coronal sections were made using a brain matrix (Agar Scientific, England). Brain sections were incubated in 2% 2,3,5triphenyltetrazolium chloride (TTC, Sigma-Aldrich, St. Louis, MO, USA) for 15 min at 37°C. The normal brain tissue was stained brick red while cerebral infarct remained pale. The infarct and total hemispheric areas of each slice were measured using Image J (ImageJ 1.5; NIH, Bethesda, MD, USA). Nissl staining was performed to calculate cerebral infarct volume and brain morphological changes at 30 d after MCAO, as previously reported [22]. Briefly, rats were perfused with ice-cold PBS and then 10% formaldehyde solution (Sigma-Aldrich, St. Louis, MO, USA). The brains were removed, fixed in 10% formaldehyde solution for another 48 h, and dehydrated using an increasing gradient, 20% to 30%, sucrose solution. Brains were coronally sliced with 20 *μ*m thickness on a freezing microtome (LM3050S; Leica Microsystems, Bannockburn, III, Germany). Before staining, sections were completely dried in a 50°C oven and immersed into a decreasing gradient, 95% Flex and 70% Flex (Sigma-Aldrich, St. Louis, MO, USA), for 1 min. Sections were then washed with distilled water for 30 seconds and incubated with Cresyl Violet (Sigma-Aldrich, St. Louis, MO, USA) for 1 min. Sections were rinsed in distilled water, dehydrated in 100% Flex, and cleared in xylene (Sigma-Aldrich, St. Louis, MO, USA). The sections were covered by slips with DPX Mountant (Sigma-Aldrich, St. Louis, MO, USA) and observed with light microscopy.

### 2.8. Evaluation of Neurodegeneration by Golgi-Cox Staining

Neurodegeneration assessed neuronal dendrites and dendritic spine degeneration via FD rapid GolgiStain^TM^ Kit according to the manufacturer recommendations. Briefly, after behavioral evaluation, rats were sacrificed, and the brain was removed, washed, and placed in Golgi-Cox sample preparation reagents at room temperature for 24 h. Brains were then kept in solution in dark conditions at room temperature for another 10 days. Coronal sections with 150 micrometers were taken onto gelatin-coated slides. The sections were treated with the staining solution followed by dehydration using gradients of ethanol (50%-100%). The sections were cleared with Xylene (Sigma-Aldrich, St. Louis, MO, USA), mounted with DPX Mountant (Sigma-Aldrich, St. Louis, MO, USA), and observed with light microscopy.

### 2.9. Western Blotting Analysis

Western blotting analysis was conducted as previously reported [34]. Right hemisphere brain tissues were collected from each rat (*n* = 5), and the protein was extracted using a protein extraction kit (sc-24948, Santa Cruz Biotechnology Inc., TX, USA) supplemented with protease inhibitor cocktail and halt phosphatase inhibitor cocktail. Equal amounts of protein sample were separated by SDS-polyacrylamide gel electrophoresis and transferred to PVDF membranes (EMD Millipore Inc., Billerica, MA, USA), which were blocked for 1 h at room temperature in TBS-T buffer supplemented with 5% nonfat dried milk. Membranes were incubated overnight at 4°C with primary antibodies followed by 1 h at room temperature with horseradish peroxidase- (HRP-) conjugated secondary antibodies (see Table 1, for details of the antibodies used). Proteins were detected using an enhanced chemiluminescent detection system (RPN2232, Amersham Bioscience, Arlington Heights, IL, USA). Band densities were quantified by the Image J software.

### 2.10. Immunohistochemical Staining

Immunohistochemical staining was performed per manufacturer guidelines. Following behavioral evaluations, rats were perfused with ice-cold PBS followed by 4% paraformaldehyde. The brains were collected and fixed in formalin and dehydrated with 20% and 30% sucrose. Next, brain samples were snap-frozen and cut into 10 *μ*m thick coronal sections using a cryostat (LM3050S; Leica Microsystems, Bannockburn, Germany). Heat-induced antigen retrieval was achieved by boiling sections for 10 min in a microwave oven in 0.01 M citrate buffer pH 6.0. Slides were blocked with endogenous peroxidase for 5 min. Then, sections were subsequently blocked for 1 h in 5% goat serum, incubated overnight at 4°C with primary antibodies, and stained for 1 h at room temperature with HRP-conjugated secondary antibodies. Protein was detected using 3-amino-9-ethylcarbazole (AEC) color developing solution (RPN2232, Amersham Bioscience, Arlington Heights, IL, USA). The sections were cleared, mounted with Gelatin glycerin (Sigma-Aldrich, St. Louis, MO, USA), and observed with light microscopy.

### 2.11. Immunofluorescence Staining

Immunofluorescence staining was performed as previously described [35]. Rats were perfused with ice-cold PBS followed by 4% paraformaldehyde. The brains were collected and fixed in formalin and dehydrated with 20% and 30% sucrose. Brain samples were snap-frozen and cut into 10 *μ*m thick coronal sections using a cryostat (LM3050S; Leica Microsystems, Bannockburn, Germany). The sections were fixed with 4% paraformaldehyde for 10 min at room temperature, washed three times in 1X Tris-buffered saline (TBS), blocked for 1 h in 5% goat serum and 0.5% Triton X-100 in 1X TBS, and incubated overnight at 4°C with primary antibodies. Finally, secondary antibodies were used for 1 h at room temperature with red, green, and blue color fluorescently labeling (Table 1). Coverslips were mounted on Super Frost Plus slides with Vectashield Hardset (Vector Labs, Burlingame, CA), and sections were visualized with a fluorescence microscope (DMi8, Leica Microsystems, Germany).

### 2.12. Macrophage Depletion with Clodronate Liposome (CLP) Injection

Macrophage depletion was induced by clodronate liposomes (CLPs, FormuMax, Sunnyvale, CA, USA). The CLPs were stored at 4°C. The original solution was used for tail vein injections every other day, according to manufacturer instructions, in the rMCAO+CLP group. Similarly, control liposomes (PBS) were injected in the rMCAO+PBS group.

### 2.13. Statistical Analysis

All data were analyzed by the Graph Pad Prism 7 software (San Diego, CA), and for parametric data, statistical significance was determined by one-way ANOVA analysis of variance with Tukey's multiple comparisons test. For nonparametric data, statistical significance was determined with a one-way ANOVA with Dunn's post hoc. A two-way ANOVA was used in neurobehaviour tests involving continuous data with multiple groups and multiple time points. Results are presented as mean ± standard deviation (SD). Differences were considered to be statistically significant with *P* < 0.05.

## 3. Results

### 3.1. Model Establishment

To evaluate the stability of the AIS model, several measurements were used including Laser Doppler flow probe, MRI-T2 maps, and TTC staining. Laser Doppler flow probe monitor showed decreased cerebral blood flow (CBF) versus baseline in both rMCAO and pMCAO groups (Additional file 2: Fig. S1a-b). MRI-T2 maps of sham and MCAO groups after 3 days of occlusion and prior to recanalization revealed no significant difference in T2 hyperintensity between rMCAO and pMCAO groups (Additional file 2: Fig. S2c-d). No significant difference was observed in infarction volume between rMCAO and pMCAO groups (Additional file 2: Fig. S2 f-e). Recanalization was confirmed by CBF and PET/CT. After recanalization, CBF increased in the rMCAO group (Additional file 3: Fig. S3 a-b). Also, the right hemisphere glucose Standardized Uptake Value (SUV) in PET/CT was increased in the rMCAO group at day 4 (1 day postrecanalization) compared to the pMCAO group (Additional file3: Fig. S2 c-d). Together, this data confirms a successful recanalization model.

### 3.2. The Effect of Delayed Recanalization on Cerebral Hemorrhage and Edema

**Figure 1:**
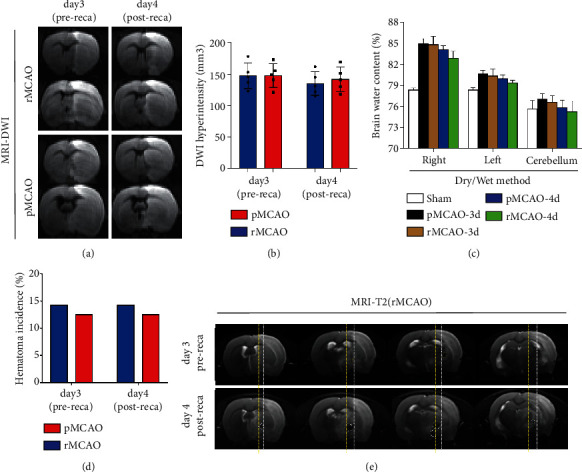
The effects of delayed recanalization on hemorrhage and edema volumes. (a) Representative image of brain edema detected via MRI-DWI scanning before and 1 day after recanalization. (b) MRI-DWI hyperintensity volume measured before and 1 day after recanalization. (c) Brain water content measured via dry/wet method in pMCAO group, rMCAO group, and 1-day recanalization. (d) Hematoma incidence was measured by calculating the occurrence of hematoma detected on MRI images. (e) Representative image of hematoma expanding after recanalization. The hypointense signal outlined on MRI-T2 images indicated acute hematoma. Yellow lines (bottom row) outline the hematoma edge after recanalization, and the white lines (top row, middle two images) outline the hematoma prior to recanalization. Error bars represent the mean ± SD. ^∗^*P* < 0.05 and ^∗∗^*P* < 0.01 (*n* = 5–8, reca: recanalization).

The conventional window for reperfusion has not been challenged due to a potential risk of ischemia/reperfusion injury and hemorrhagic transformation [7, 8].  Figure 1(a) is representative of MRI-DWI hyperintensity consistent with restricted diffusion on both rMCAO and pMCAO prior to recanalization and following recanalization. Quantified mean and standard deviation of DWI hyperintensity (mm^3^) stratified by group are shown in Figure 1(b). Compared to the pMCAO group, there was no significant difference detected in DWI hyperintensity volume in the rMCAO group *P* > 0.05. In Figure 1(c), brain water content (%) was not statistically different among MCAO groups (*P* > 0.05). Hematoma incidence (%) was not statistically different among MCAO groups (Figure 1(d); *P* > 0.05). However, qualitatively, the hematoma volume observed via MRI appeared to be larger following recanalization if it existed prerecanalization (Figure 1(e)).

### 3.3. Delayed Recanalization Improves Long-Term Neurological Outcome in Selected Rats

Long-term neurological outcome was evaluated by measuring sensorimotor function, plasticity, and memory. Beam walking and neurological tests were evaluated at 3, 10, and 33 days (prerecanalization, one week after recanalization, and one month after recanalization, respectively). Rats with neurological scores < 6 at day 3 were excluded. In the rMCAO group, we excluded 3, 0, and 2 animals from their designated endpoint: days 3, 10, or 33, respectively. In the pMCAO group, we excluded 1, 1, and 2 animals from their designated endpoint: days 3, 10, or 33, respectively. Details of the exclusion number are provided in Table S1. Although both beam walking and neurological scale scores trended toward improved outcome, there was no statistical difference among groups (Additional file 4: Fig. S4 a-b). Compared to the pMCAO group, no statistical difference was detected in the rMCAO group (Additional file 4: Fig. S4 c-d). During the probe test, compared to the pMCAO group, probe quadrant duration and passing times trended toward improvement in the rMCAO group, although no statistical difference was detected (Additional file 4: Fig. S4 e-f).

**Figure 2:**
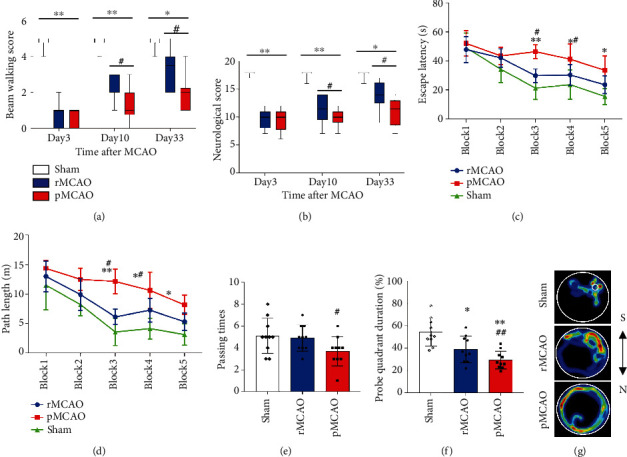
Delayed recanalization improves long-term neurological outcome. (a) Beam walking balance. (b) Neurological score. (c) Escape latency in the water maze test. (d) Path length in the water maze test. (e) Probe quadrant passing times in the water maze test. (f) Probe quadrant duration in the water maze test. (g) Representative heatmaps of water maze tracking. Error bars represent median (range) ± interquartile range (Q1 to Q3) for (a, b). Error bars represent the mean ± SD for (c–f). ^∗^*P* < 0.05 and ^∗∗^*P* < 0.01 vs. sham group; ^#^*P* < 0.05 and ^##^*P* < 0.01 vs. rMCAO group (*n* = 10).

Data are presented as the median (range) ± interquartile range (Q1 to Q3) for beam walking scores at days 3, 10, and 33 are shown in Figure 2(a). Compared to the sham group, beam walking scores were reduced in all MCAO groups (*P* < 0.05). Compared to the pMCAO group, beam walking scores were improved in the rMCAO group at both days 10 and 33 (*P* < 0.05). Median (range) ± interquartile range (Q1 to Q3) of neurological score after MCAO at days 3, 10, and 33 are shown in Figure 2(b). Compared to the sham group, neurological score for both rMCAO and pMCAO groups was decreased (*P* < 0.05). Compared to the pMCAO group, neurological score improved in the rMCAO group at both 10 and 33 days (*P* < 0.05).

Mean and standard deviation for escape latency (sec) are shown in Figure 2(c). Compared to the sham group, escape latency was increased in blocks 3, 4, and 5 in pMCAO and rMCAO groups. Compared to the rMCAO group, escape latency was increased in blocks 3 and 4 in the pMCAO group (*P* < 0.05). Mean and standard deviation for path length (meters) are shown in Figure 2(d). Compared to the sham group, path length increased in blocks 3, 4, and 5 in pMCAO and rMCAO groups (*P* < 0.05). Compared to the rMCAO group, the path length was increased in blocks 3 and 4 in the pMCAO group (*P* < 0.05).

Mean and standard deviation of passing times are shown for sham, rMCAO, and pMCAO groups in Figure 2(e). Compared to the rMCAO group, passing time was decreased in the pMCAO group (*P* < 0.05). Mean and standard deviation of probe quadrant duration (%) are shown for sham, rMCAO, and pMCAO groups in Figure 2(f). Compared to the sham group, probe quadrant duration for both rMCAO and pMCAO groups was decreased (*P* < 0.05). Compared to the rMCAO group, time spent in the probe quadrant was decreased in the pMCAO group (*P* < 0.05).  Figure 2(g) shows representative examples of water maze tracking for sham, rMCAO, and pMCAO groups.

### 3.4. Delayed Recanalization Reduces Neurodegeneration

After the long-term neurological test, tissue loss was evaluated via Nissl staining. Compared to the pMCAO group, the infarct volume was reduced in the rMCAO group. Normal neuronal arrangement was also present in the rMCAO group (Additional file 5: Fig. S5a-d). Representative regions of interest for the Nissl staining in the cortex and hippocampal regions revealed increased loss of tissue and abnormal neurons in the pMCAO group compared to the rMCAO group, especially in the CA2 hippocampal region (Additional file 5: Fig. S5e).

**Figure 3:**
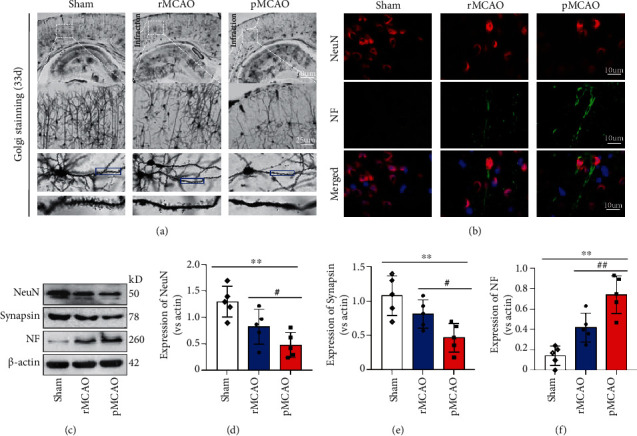
Histological evaluation of neurodegeneration after delayed recanalization. (a) Representative images of Golgi staining. Neuron, dendrites and spinous process numbers, and arrangement were significantly altered in MCAO groups compared to the sham group. Delayed recanalization reversed these changes in the rMCAO group compared to the pMCAO group. (b) Immunofluorescence staining showed that delayed recanalization dramatically reduced neurofilament protein expression. (c) Western blotting analysis showed that delayed recanalization reduced neurofilament protein expression while increasing the expression of NeuN and synapsin. (d–f) Quantification of related protein expressions in each group. Error bars represent the mean ± SD. ^∗^*P* < 0.05 and ^∗∗^*P* < 0.01 vs. sham group; ^#^*P* < 0.05 and ^##^*P* < 0.01 vs. rMCAO group (*n* = 5).

Figure 3(a) shows representative examples of Golgi-Cox staining in sham, pMCAO, and rMCAO groups. Golgi-Cox staining showed that pMCAO rats exhibited reductions in bilateral spine density and dendritic arborization compared to rMCAO and sham groups.  Figure 3(b) shows representative of neurofilament heavy chain (NF) immunoreactivity in neurons in sham, pMCAO, and rMCAO groups. Qualitatively, NF immonoreactivity was increased in the pMCAO group compared to rMCAO and sham groups.  Figure 3(c) shows representative Western blot for NeuN, synapsin, NF, and *β*-actin in sham, rMCAO, and pMCAO groups.  Figure 3(d) quantifies Western blot NeuN expression. Compared to the sham group, NeuN expression was decreased in rMCAO and pMCAO groups (*P* < 0.05). Compared to the rMCAO group, NeuN expression was further decreased in the pMCAO group (*P* < 0.05).  Figure 3(e) quantifies Western blot synapsin expression. Compared to the sham group, synapsin expression was decreased in rMCAO and pMCAO groups (*P* < 0.05). Compared to the rMCAO group, synapsin expression was further decreased in the pMCAO group (*P* < 0.05).  Figure 3(f) quantifies Western blot NF expression. Compared to the sham group, NF expression was increased in rMCAO and pMCAO groups (*P* < 0.05). Compared to the rMCAO group, NF expression was increased in the pMCAO group (*P* < 0.05).

### 3.5. Delayed Recanalization Increases Angiogenesis and Microvasculature Stabilization

Cerebrovascular remodeling to maintain the “neurovascular unit” is a fundamental process governing neuronal repair in response to ischemia. Angiogenesis can significantly reduce neurodegeneration by supporting the metabolic demands for neurons, increasing neuronal plasticity, and promoting neural progenitor cell migration/differentiation [32, 33].

**Figure 4:**
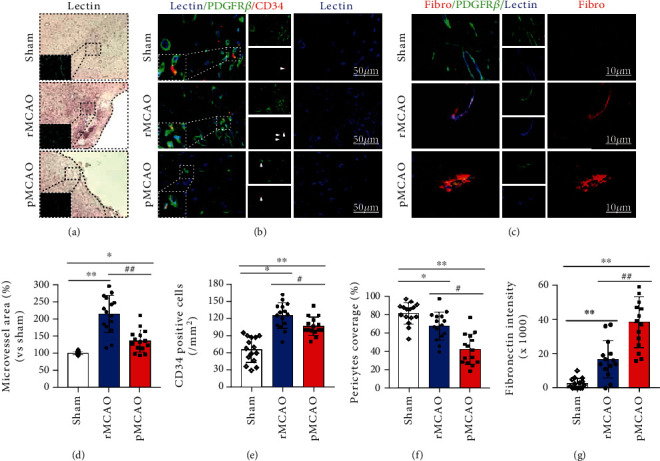
Angiogenesis after delayed recanalization. (a) Representative image of microvessels stained with lectin in sham, rMCAO, and pMCAO groups. (b) Representative immunohistochemical staining for lectin, PDGFR*β*, and CD34. Angiogenesis is depicted by cells expressing colocalization of PDGFR*β* and CD34 markers around the endothelial cells. (c) Representative immunohistochemical staining for fibronectin, PDGFR*β*, and CD34; BBB leakage is depicted by fibronectin expression around microvessels. (d) Quantification of microvessel area in each group. (e–g) Quantification of CD34-positive cells, pericyte coverage, and BBB leakage in each group. Error bars represent the mean ± SD. ^∗^*P* < 0.05 and ^∗∗^*P* < 0.01 vs. sham group; ^#^*P* < 0.05 and ^##^*P* < 0.01 vs. rMCAO group (*n* = 5).

Figure 4(a) shows representative images of endothelial cells visualized by staining for lectin in sham, rMCAO, and pMCAO groups. CD34 is selectively expressed on the surface of mammalian hematopoietic stem/progenitor cells. CD34-positive endothelial progenitor cells are important in the formation of new blood vessels in the ischemic brain area, contributing to angiogenesis in the acute stage of stroke and neuroregeneration [36, 37]. Therefore, CD34 is widely used as a marker of angiogenesis. Pericyte cells, also known as Rouget cell and parietal cell, surround endothelial cells in capillaries and veins. They are embedded in the basement membrane of capillary endothelial cells and interact with endothelial cells through physical contact and paracrine signals. Microvasculature formation, maturation, and stabilization were measured by immunofluorescence staining of CD34-positive endothelial progenitor cells, PDGFR*β*^−^positive pericyte coverage, and fibronectin leakage.

Figure 4(b) shows representative immunohistochemical staining for lectin, PDGFR*β*, and CD34; pericyte coverage is depicted by cells expressing colocalization of PDFR*β* and CD34 markers around the endothelial cells.  Figure 4(c) shows representative immunohistochemical staining for fibronectin, PDGFR*β*, and CD34; BBB leakage is depicted by fibronectin expression.  Figure 4(d) quantifies microvessel coverage in sham, rMCAO, and pMCAO groups. Compared to the sham group, microvessel coverage was increased in pMCAO and rMCAO groups (*P* < 0.05). Compared to the pMCAO group, microvessel coverage was increased in the rMCAO group (*P* < 0.05).  Figure 4(e) quantifies CD34-positive cells in sham, rMCAO, and pMCAO groups. Compared to the sham group, CD34-positive cells are increased in both rMCAO and pMCAO groups (*P* < 0.05). Compared to the rMCAO group, CD34 cells are further decreased in the pMCAO group (*P* < 0.05).  Figure 4(f) quantifies the expression of pericyte coverage in sham, rMCAO, and pMCAO groups. Compared to the sham group, pericyte coverage was reduced in rMCAO and pMCAO groups (*P* < 0.05). Compared to the pMCAO group, pericyte coverage was increased in the rMCAO group (*P* < 0.05).  Figure 4(g) quantifies vessel leakage via the expression of fibronectin in sham, rMCAO, and pMCAO groups. Compared to the sham group, fibronectin expression and intensity are increased in rMCAO and pMCAO groups (*P* < 0.05). Compared to the pMCAO group, fibronectin expression and intensity were reduced in the rMCAO group (*P* < 0.05).

### 3.6. Angiogenesis after Delayed Recanalization Is Associated with Macrophage Infiltration

Macrophages play a predominant role in inflammatory responses, and depending on phenotypic plasticity, they may promote tissue repair and angiogenesis [27, 38]. Evidence supports that sanguineous exposure to macrophages may promote tissue repair in ischemic tissue via accelerated angiogenesis [26].

**Figure 5:**
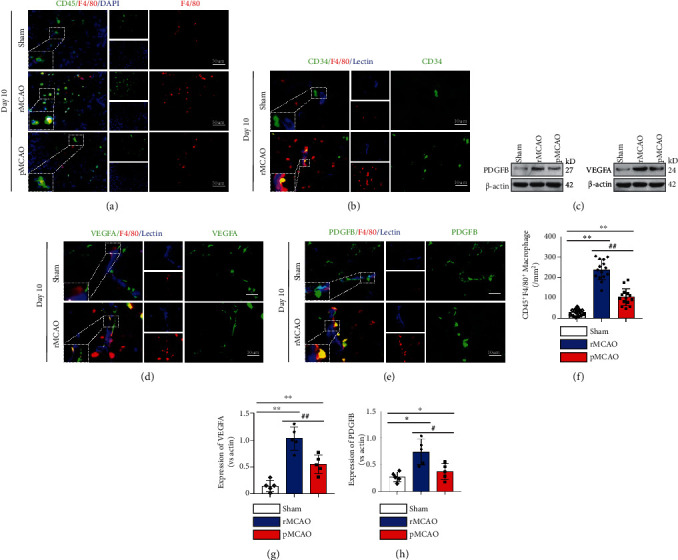
Macrophage infiltration and angiogenesis analysis. (a) Representative image of macrophage staining with CD45 and F4/80. (b) Representative immunohistochemical staining for F4/80-positive macrophage and CD34-positive endothelial progenitor cells. (c) Western blotting analyses of VEGFA and PDGFB expressions. (d) Representative images for VEGFA and F4/80 immunohistochemical staining. (e) Representative immunohistochemical staining for PDGFB and F4/80. (f) Quantification of macrophage infiltration in each group. (g) Quantification of VEGFA expression. (h) Quantification of VEGFA expression. Error bars represent the mean ± SD. ^∗^*P* < 0.05 and ^∗∗^*P* < 0.01 vs. sham group; ^#^*P* < 0.05 and ^##^*P* < 0.01 vs. rMCAO group.

Figure 5(a) shows representative F4/80 (macrophages) and CD45 (B lymphocytes and a subset of T-lymphocytes and NK cells) in sham, pMCAO, and rMCAO groups.  Figure 5(b) shows representative CD34 (immature hematopoietic precursor cells), F4/80 (macrophages), and lectin (microvasculature) in sham, pMCAO, and rMCAO groups.  Figure 5(c) shows representative images of Western blot of PDGFR*β* and VEGFA in sham, rMCAO, and pMCAO groups at 10 days after infarction (7 days after recanalization).  Figure 5(d) shows representative immunohistochemistry of vascular endothelial growth factor A (VEGFA), F4/80, and lectin at 10 days after infarction in sham and rMCAO groups.  Figure 5(e) shows representative immunohistochemistry images of PDGFR*β*, F4/80, and lection at 10 days after infarction in sham and rMCAO groups.  Figure 5(f) quantifies the number of macrophages (colocalization of CD45 with F4/80) in sham, pMCAO, and rMCAO groups. Compared to the sham group, the number of macrophages was increased in pMCAO and rMCAO groups (*P* < 0.05). Compared to the pMCAO group, the number of macrophages was increased in the rMCAO group (*P* = 0.05).  Figure 5(g) quantifies the expression of VEGFA in sham, rMCAO, and pMCAO groups at 10 days after infarction. Compared to the sham group, VEGF was elevated in rMCAO and pMCAO groups (*P* < 0.05). Compared to the rMCAO group, VEGF was decreased in the pMCAO group (*P* < 0.05).  Figure 5(h) quantifies the expression of PDGF*β* in sham, rMCAO, and pMCAO at 10 days after infarction. Compared to the sham group, the expression of PDGF*β* was increased in rMCAO and pMCAO groups (*P* < 0.05). Compared to the pMCAO group, the expression of PDGF*β* was increased in the rMCAO group (*P* < 0.05).

### 3.7. Macrophage Depletion Reduces Angiogenesis after Delayed Recanalization

Clodronate liposomes (CLPs) can use mechanisms such as endocytosis by macrophages to bring and release membrane-impermeable chlorophosphoric acid (clodronate) into the cell. It can also trigger the apoptotic pathways in macrophages, thereby reducing the number of circulating macrophages.

**Figure 6:**
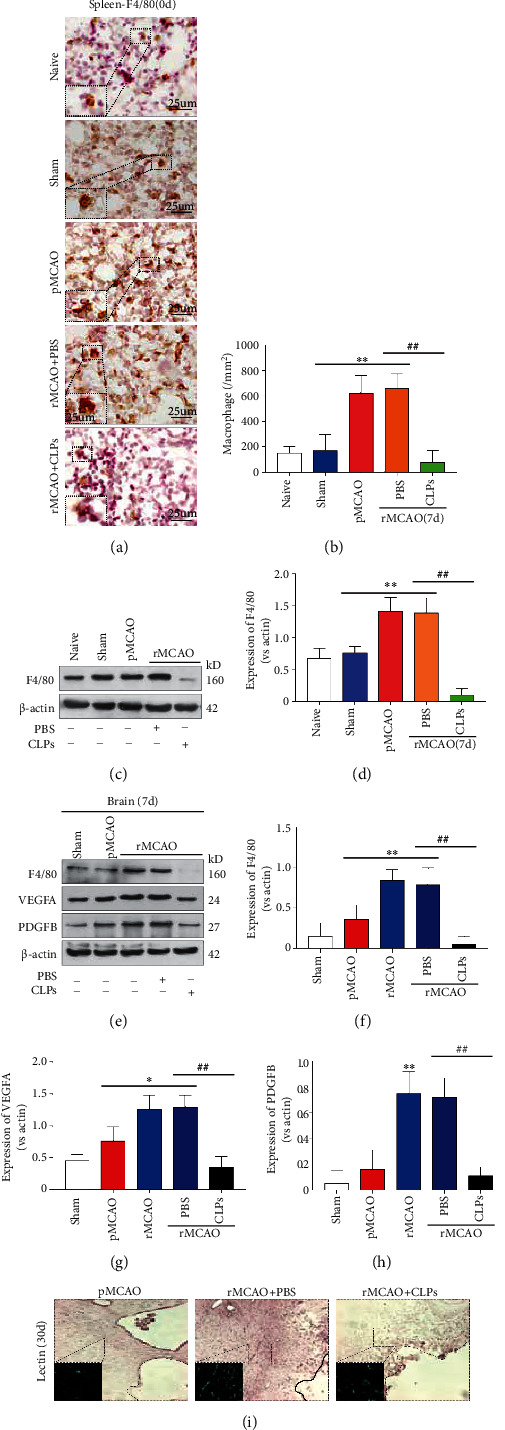
Macrophage depletion reduces angiogenesis after delayed recanalization. (a) Representative images of macrophages via F4/80 immunohistochemical staining in the spleen. (b) Quantification of macrophage in each group. (c) Western blotting analysis of macrophage marker F4/80 expression. (d) Quantification of macrophage marker F4/80 expression in spleen. (e) Western blotting analyses of macrophage, neuroinflammation, and angiogenesis markers. (f–h) Quantification of related proteins in each group. (l) Representative images of microvessel density in the peri-infarction area visualized by immunohistochemical staining with lectin. Error bars represent the mean ± SD. ^∗^*P* < 0.05 and ^∗∗^*P* < 0.01 vs. sham group; ^#^*P* < 0.05 and ^##^*P* < 0.01 vs. rMCAO group; ^&^*P* < 0.05 and ^&&^*P* < 0.01 vs. rMCAO+PBS (*n* = 5).

Figure 6(a) shows representative macrophage staining of the spleen in naive, sham, pMCAO, rMCAO+PBS, and rMCAO+CLP groups. No significant difference in macrophage number was detected between naive and sham groups. Compared to the sham group, the number of macrophages increased in the pMCAO and rMCAO groups (*P* < 0.05). Compared to the rMCAO group, the number of macrophages was decreased in the rMCAO+CLP group (*P* < 0.05).  Figure 6(c) shows representative immunoblots of the spleen. Compared to the sham group, the expression of F4/80 was increased in pMCAO and rMCAO groups and decreased in the rMCAO+CLP group. Compared to the rMCAO group, the expression of F4/80 was reduced.

Figure 6(e) shows representative immunoblots on the ipsilateral hemisphere for F4/80, IL1*β*, VEGFA, PDGF*β*, CD34, and PDGFR*β* at 10 days following infarction. Compared to the sham group, the expression of IL1*β*, VEGFA, PDGF*β*, and CD34 was increased in pMCAO, rMCAO, and rMCAO+PBS groups, whereas the expression of PDGFR*β* was decreased in pMCAO, rMCAO, and rMCAO+PBS groups (*P* < 0.05; Figures 6(f)–6(h)). Compared to the rMCAO+PBS group, the expression of F4/80, IL1*β*, VEGFA, PDGF*β*, CD34, and PDGFR*β* was reduced in the rMCAO+CLPs group (*P* < 0.05; Figures 6(f)–6(h)).  Figure 6(i) shows representative lectin peri-infarct staining at 33 days following infarction in rMCAO and rMCAO+CLP groups; qualitatively, lectin expression was reduced in the rmCAO+CLP group compared to the rMCAO group.

### 3.8. Macrophage Depletion Reduces Neurological Recovery following Delayed Recanalization

**Figure 7:**
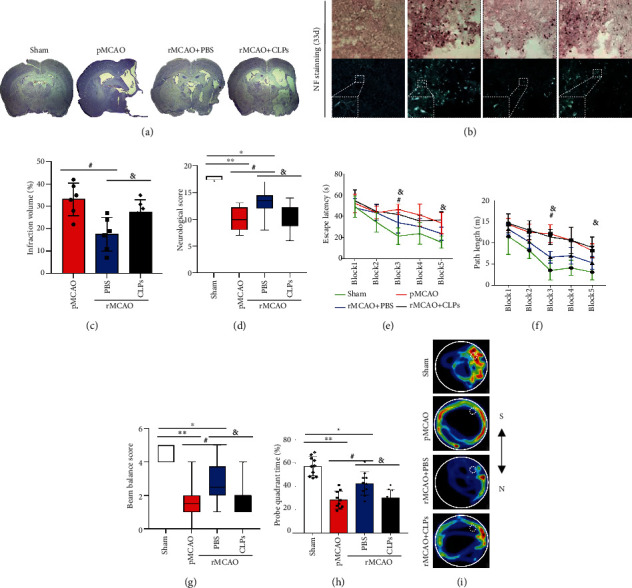
Macrophage depletion reduces neurological recovery. (a) Brain tissue loss visualized by coronal Nissl staining for sham, pMCAO, rMCAO+PBS, and rMCAO+CLP groups. (b) Representative images of neurofilament protein staining in each group. (c) The infarction volume was calculated from Nissl stained sections. (d) Neurological scores acquired in each group. (e) Escape latency in the water maze test. (f) Path length in the water maze test. (g) Beam walking balance score. (h) Probe quadrant duration in the water maze test. (i) Representative examples of water maze tracking. Error bars represent median (range) ± interquartile range (Q1 to Q3) for (d, g). Error bars represent the mean ± SD for (c, e, f, h). ^∗^*P* < 0.05 and ^∗∗^*P* < 0.01 vs. sham group; ^#^*P* < 0.05 and ^##^*P* < 0.01 vs. rMCAO group; ^&^*P* < 0.05 and ^&&^*P* < 0.01 vs. rMCAO+PBS (*n* = 10–15).

Figure 7(a) shows representative coronal Nissl staining for sham, pMCAO, rMCAO+PBS, and rMCAO+CLP groups.  Figure 7(b) shows representative NF staining of the peri-infarction region for sham, pMCAO, rMCAO+PBS, and rMCAO+CLP groups. The infarction volume was decreased in the rMCAO+PBS group compared to the pMCAO group, whereas it was increased in the rMCAO+CLP group compared to the pMCAO group (*P* < 0.05; Figure 7(c)). Compared to the sham group, neurological and beam balance scores were reduced in pMCAO, rMCAO+PBS, and rMCAO+CLP groups (*P* < 0.05; Figures 7(d) and 7(g)). Compared to the rMCAO+PBS group, neurological and beam balance scores were decreased in pMCAO and rMCAO+CLP groups (*P* < 0.05; Figures 7(d) and 7(g)). Mean and standard deviation for escape latency (sec) are shown in Figure 7(e). Mean and standard deviation for path length are shown in Figure 7(f). Compared to the sham group, the escape latency and path length were increased for blocks 3 and 5 in pMCAO and rMCAO+CLP groups (*P* < 0.05). Compared to the rMCAO+PBS group, the escape latency and path length were increased in block 3 in pMCAO and rMCAO+CLP groups (*P* < 0.05). Compared to the sham group, the probe quadrant time (%) was decreased in all MCAO groups (*P* < 0.05; Figures 7(h)). Compared to the rMCAO+PBS group, the probe quadrant time (%) was decreased in pMCAO and rMCAO+CLP groups (*P* < 0.05).  Figure 7(i) shows representative examples of water maze tracking for sham, pMCAO, rMCAO+PBS, and rMCAO+CLP groups.

## 4. Discussion

During the last two decades, basic science stroke research has investigated new therapies to promote neurological recovery and lower patient mortality. Although a significant number of neuroprotectants (>1000) have shown efficacy in animal models, translational trials have failed such that rt-PA remains the only FDA-approved drug [39].

Ischemic stroke can roughly be divided into three phases: acute, subacute, and delayed phase. During the acute phase, abrupt disruption of cerebral blood flow triggers energy failure followed by irreversible cell death in the ischemic core; however, around the core, penumbral tissue that meets a survival but not a functional energy demand is the target for treatment intervention. During the subacute phase that occurs several hours after onset of ischemia, irreversible tissue damage converts the penumbra into the expanding ischemic core. If reperfusion is not achieved in the subacute phase, a delayed phase (days to weeks after symptom onset) may occur in which ischemic injury is further exacerbated by secondary oxidative stress, brain edema, neuroinflammation, and other associated and relevant deleterious molecular mechanisms [40, 41]. The driving principle behind reperfusion therapy is the “penumbra.” If large enough, a penumbra ensures salvageable parenchyma. Specifically, if cerebral blood flow meets a survival threshold, ischemic damage is mitigated; however, irreversible tissue damage occurs when blood flow is decreased below a certain percent of preocclusion values [42, 43]. Thus, the peri-infarct area with a perfusion range between reversible and irreversible thresholds (about 25%-50% of preocclusion values) is typically described as “penumbra” [44, 45].

The majority of patients admitted outside of the progressively expanding but still limited reperfusion window could significantly benefit from delayed recanalization given sufficient penumbra volume. In support of this hypothesis, one study recently reanalyzed the data from the DEFUSE 3 study and suggested that about 20% of ischemic stroke patients with LVO who are not treated with thrombectomy had a persistent mismatch of up to 48 hours [20]. Subsequently, numerous case reports suggest a functional association between delayed blood flow restoration and neurological outcome conditional to imaging mismatch beyond 3 days from symptom onset [46, 47]. In this study, using ischemia durations of 3 days, we validated the effect of delayed recanalization on neurological deficits and the underlying mechanisms driving these effects in a rat MCAO model. In spite of observed persistent decline of neurologic function in both delayed recanalization and permanent ischemia groups, delayed recanalization at 3 days improved neurobehavioral outcomes compared to nonreperfused rats without significant increase in mortality rate or incidence of intracerebral hemorrhage.

Cerebrovascular remodeling to maintain the “neurovascular unit” is a fundamental process governing neuronal repair in response to ischemia. In similar fashion, reperfusion sustains the integrity and health of the neurovascular unit by supporting the metabolic demands for neurons, increasing neuronal plasticity, and promoting neural progenitor cell migration/differentiation [48]. Cerebrovascular regeneration and angiogenesis are thus becoming promising therapeutic strategies, and given their importance, we investigated the role of angiogenesis in delayed recanalization after stroke. After recanalization, the number of CD34-positive endothelial progenitor cells and lectin-positive microvessel density in the peri-infarct area were increased compared to nonreperfused rats.

In this study, the microvessel density in the peri-infarction area congruently increased in both pMCAO and rMCAO groups at 33 days following ischemia, but a higher density was observed in the rMCAO group. In this report, we detected increased neurodegeneration in both the pMCAO and rMCAO groups through Golgi staining, neurofilament heavy chain staining, and Western blot analysis. Nonetheless, the extent of neurodegeneration was mitigated in the rMCAO group; the neuronal number and dendrite intensity and spines were considerably higher in the rMCAO group. Concurrently, neurofilament expression was reduced in the rMCAO group compared to the pMCAO group. We propose that favorable outcomes in the rMCAO group are in part due to higher vascular maturation with increased pericyte coverage and less blood brain barrier (BBB) permeability relative to the pMCAO group. Accordingly, studies have shown increased pericyte coverage on endothelial cells to be associated with significantly less BBB permeability in stroke animal models [49]. Presumably, a number of reperfusion-dependent peripheral circulation factors and/or cells are necessary for vascular regeneration in the peri-infarction regions.

Revascularization after ischemic stroke promotes angiogenesis via cellular and molecular pathways. Vascular endothelial growth factor (VEGF) and platelet-derived growth factor (PDGF) signaling pathways are reported as critical factors that promote angiogenesis in ischemic tissue [50–53]. While VEGF initiates vessel regeneration, the expression of PDGF promotes pericyte recruitment and maturation of naive vessels [54]. In the current study, the expression of VEGF-A and PDGF-B was significantly increased in both the pMCAO and rMCAO groups at 7 days after ischemic stroke; both were increased in the rMCAO group compared to the pMCAO group.

Further assessment revealed that VEGF and PDGF were predominantly colocalized with F4/80-positive macrophages that surrounded the vessels in the peri-infarcted area. The majority of investigators agree that macrophages are polarized toward M1 and M2 phenotypes, M1 producing proinflammatory cytokines, presenting antigens, and producing reactive oxygen species, and M2 producing immune tolerance, tissue remodeling (VEGF and PDGF), and neuroprotection [55, 56]. In sum, favorable outcomes in stroke models are dependent on sanguineous exposure to macrophages.

Traditionally, one week after stroke onset, inflammatory processes, such as the activation of macrophages, play an influential role in stroke severity and functional outcomes [57]. In ischemic models, studies suggest that macrophage recruitment promotes tissue repair via an angiogenic process [58]. Similarly, we observed an increase in the number of macrophages in the peri-infarction area at 7 days following recanalization,. To elucidate the role of macrophages in angiogenesis, we used Clodronate liposomes in a pharmacological approach that depleted macrophages from the circulation. We observed a paradoxical result; specifically, while macrophage depletion leads to reduced activation of inflammatory pathways in the brain, worsened neurological outcomes were ironically observed in macrophage depleted groups, speculatively through reduced angiogenesis and decreased pericyte coverage. Taken together, a potential mechanism unfolds in which early infiltrating macrophages in the setting of delayed recanalization sustain vessel function in the peri-infarct area, therefore attenuating neurodegeneration and improving functional recovery after stroke. Caveat, the specific molecular mechanism of macrophage recruitment and activation of angiogenesis after ischemic infarct remains incompletely characterized. Furthermore, our research focuses specifically on the infarct location, but the effect of infarct is further reaching than the local infarct zone. Specifically, long-term sequela of ischemic infarct includes damage to the associated white matter track(s), i.e., Wallerian degeneration. Further research may likely reveal unique and/or overlapping benefits of macrophage recruitment for protection against white matter injury. Even so, some of our observed benefits may very well be related to improved protection against white matter injury.

Several limitations are present in this study. First, given that the prevalence of stroke increases with age, our young animal models may poorly generalize to the older populations. Comorbidities also increase with age, including hypertension and diabetes, which are known to strongly influence the efficacy of delayed recanalization. Our study did not evaluate age-related effects on stroke and recanalization. Our experiment did not use female rats and was male biased. Also, the rMCAO model clinically represents mechanical thrombectomy (abrupt reperfusion) rather than fibrinolysis (slower reperfusion). Lastly, although our results show delayed recanalization improves neurological outcome, neurological testing in animal models has inherently lower sensitivity compared to translational research.

In summary, the current study supports that increased macrophage infiltration promotes neurological recovery after recanalization at three days after ischemic infarction via increased angiogenesis and reduced neurodegeneration. We provide evidence that delayed recanalization (>24 hours) results in favorable outcomes when compared to nonrecanalized groups. As the window of reperfusion expands, further research is needed on delayed recanalization and its impact on stroke pathophysiology.

## Figures and Tables

**Table 1:** Details of antibodies used.

Antibody	Source	Dilution
NeuN (rabbit)	Abcam (ab177487)	1 : 2000 (WB), 1 : 200 (IF)
NeuN (mouse)	Abcam (ab104224)	1 : 2000 (WB), 1 : 200 (IF)
Synapsin (rabbit)	Abcam (ab64581)	1 : 1000 (WB)
Neurofilament (rabbit)	Abcam (ab207176)	1 : 1000 (WB), 1 : 200 (IF, IHC)
*β*-Actin (mouse)	Santa Cruz (sc-47778)	1 : 3000 (WB)
Lectin	VectorLab (B-1175-1)	1 : 500 (IHC), 1 : 200 (IF)
PDGFR*β* (mouse)	Abcam (ab69506)	1 : 2000 (WB), 1 : 200 (IF)
CD34 (rabbit)	Abcam (ab81289)	1 : 200 (IF)
CD45 (rabbit)	Abcam (ab40763)	1 : 1000 (WB), 1 : 200 (IF)
Fibronectin (rabbit)	Abcam (ab268020)	1 : 100 (IF)
F4/80 (rat)	Abcam (ab6640)	1 : 1000 (WB), 1 : 200 (IF, IHC)
PDGFB (rabbit)	Abcam (ab23914)	1 : 1000 (WB), 1 : 200 (IF)
VEGFA (rabbit)	Abcam (ab39250)	1 : 1000 (WB), 1 : 200 (IF)
HRP-goat anti-mouse	Santa Cruz (sc-2973)	1 : 4000 (WB)
HRP-goat anti-rabbit	Santa Cruz (sc-2357)	1 : 4000 (WB)
HRP-goat anti-rat	Santa Cruz (sc-362293)	1 : 4000 (WB)
AMCA-streptavidin	VectorLab (SA-5008-1)	1 : 200 (IF)
Alexa Fluor 488 goat anti-rabbit	Jackson (111-545-144)	1 : 200 (IF)
Alexa Fluor 568 goat anti-mouse	Jackson (115-545-166)	1 : 200 (IF)
Alexa Fluor 488 donkey anti-mouse	Jackson (715-545-020)	1 : 200 (IF)
Alexa Fluor 594 goat anti-rabbit	Jackson (111-545-003)	1 : 200 (IF)
Alexa Fluor 594 goat anti-rat	Jackson (112-585-003)	1 : 200 (IF)

WB: Western blot; IHC: immunocytochemistry; IF: immunofluorescence.

## Data Availability

All data are available upon request.

## Supplementary Materials

Supplementary Materials Supplementary Materials 1 Experiment design. Experiment I: experiment I was designed to confirm the effect of delayed recanalization on neurodegeneration after AIS. Sham, rMCAO, and pMCAO groups were evaluated in experiment I. Neurological outcomes, angiogenesis, and neurodegeneration were evaluated after delayed recanalization. Experiment II: experiment II was designed to confirm the effect of macrophage infiltration on angiogenesis after delayed recanalization. Five groups, naive, sham, pMCAO, rMCAO, rMCAO+PBS, and rMCAO+CLP groups, were established in experiment II. Neurological outcomes, angiogenesis, and neurodegeneration were evaluated after delayed recanalization. Neurological outcomes, macrophage infiltration, angiogenesis, and neurodegeneration were evaluated after delayed recanalization. Detailed experimental design is listed in the supplementary material (Additional file 1: Figure S1 and Table. S1). Additional file 1. Table S1: detailed information on rat usage. PS: ^∗^animals used for MRI scanning; ^#^animals used for PET/CT scanning. 1. Supplementary figure. Additional file 1. Figure S1: schematic illustration of the experimental design. Additional file 2. Fig S2: data for model establishment. (a) Representative image of CBF via laser Doppler flow probe during filament insertion in MCAO model establishment. (b) CBF measured via laser Doppler flow probe in pMCAO and rMCAO groups before recanalization. (c) Representative images of infarct volume via MRI-T2 scanning. (d) T2 hyperintensity measured in pMCAO and rMCAO groups before recanalization. (e) Infarct volume measured via TTC staining in pMCAO and rMCAO groups before recanalization. (f) Representative images of infarct volume via TTC staining for model establishment. Error bars represent the mean ± SD. *N* ≥ 3, ^∗^*P* < 0.05; ^∗∗^*P* < 0.01. Additional file 3. Fig S3: recanalization evaluation. (a) When recanalization was induced by filament withdrawing, CBF gradually increased in the rMCAO group. (b) CBF measured via laser Doppler flow probe in pMCAO and rMCAO groups before and after recanalization. (c) Representative image of cerebral glucose uptake in right hemisphere via PET-CT scanning for recanalization confirming. (d) The right hemisphere glucose SUV in PET/CT scanning was significantly increased at 1 day after recanalization in the rMCAO-1 d group compared to the pMCAO group. Error bars represent the mean ± SD. ^∗^*P* < 0.05; ^∗∗^*P* < 0.01; ^#^*P* < 0.05, *n* = 5. Additional file 4. Fig S4: long-term neurological outcome after delayed recanalization. (a) Beam walking balance. (b) Neurological score. (c) Escape latency in the Morris water maze test. (d) Path length in the Morris water maze test. (e) Probe quadrant passing times in the Morris water maze test. (f) Probe quadrant duration in the water maze test. (g) Representative heatmaps of water maze tracking. Error bars represent median (range) ± interquartile range (Q1 to Q3) for (a, b). Error bars represent the mean ± SD for (c, d, f, g). Additional file 4. Fig S5: brain morphology changes after delayed recanalization. (a) Brain tissue loss via eye view of brain specimen and Nissl staining. (b) Infarction volume measured via Nissl staining. (c) Regions of interest viewed via Nissl staining. In the rMCAO group, brain tissue loss on eye view and Nissl staining was significantly reduced compared to the pMCAO group. (d) Observation under microscope shows the cortical structure of the brain tissue to be more clearly visualized in the rMCAO group. Neurons were also more neatly arranged. A distinction between the cortex and deep brain tissue is more easily drawn in the rMCAO group when compared to the pMCAO group. (e) Magnified images in the cortex, and hippocampus CA1, CA2, and CA3.
