# Supplementary material for: Macrophage Infiltration Reduces Neurodegeneration and Improves Stroke Recovery after Delayed Recanalization in Rats
Source: Oxid Med Cell Longev. 2022 Aug 17;2022:6422202. doi: 10.1155/2022/6422202 (PMC9402313; doi:10.1155/2022/6422202)
Supplement: Supplementary Materials — 1 Experiment design. Experiment I: experiment I was designed to confirm the effect of delayed recanalization on neurodegeneration after AIS. Sham, rMCAO, and pMCAO groups were evaluated in experiment I. Neurological outcomes, angiogenesis, and neurodegeneration were evaluated after delayed recanalization. Experiment II: experiment II was designed to confirm the effect of macrophage infiltration on angiogenesis after delayed recanalization. Five groups, naive, sham, pMCAO, rMCAO, rMCAO+PBS, and rMCAO+CLP groups, were established in experiment II. Neurological outcomes, angiogenesis, and neurodegeneration were evaluated after delayed recanalization. Neurological outcomes, macrophage infiltration, angiogenesis, and neurodegeneration were evaluated after delayed recanalization. Detailed experimental design is listed in the supplementary material (Additional file 1: Figure S1 and Table. S1). Additional file 1. Table S1: detailed information on rat usage. PS: ∗animals used for MRI scanning; #animals used for PET/CT scanning. 1. Supplementary figure. Additional file 1. Figure S1: schematic illustration of the experimental design. Additional file 2. Fig S2: data for model establishment. (a) Representative image of CBF via laser Doppler flow probe during filament insertion in MCAO model establishment. (b) CBF measured via laser Doppler flow probe in pMCAO and rMCAO groups before recanalization. (c) Representative images of infarct volume via MRI-T2 scanning. (d) T2 hyperintensity measured in pMCAO and rMCAO groups before recanalization. (e) Infarct volume measured via TTC staining in pMCAO and rMCAO groups before recanalization. (f) Representative images of infarct volume via TTC staining for model establishment. Error bars represent the mean ± SD. N ≥ 3, ∗P < 0.05; ∗∗P < 0.01. Additional file 3. Fig S3: recanalization evaluation. (a) When recanalization was induced by filament withdrawing, CBF gradually increased in the rMCAO group. (b) CBF measured [file 6422202.f1.zip › Supplamental Table 1 (1).docx]

| **Experiments** | **Time** | **Groups** | **PET/TTC** | **MRI &**  **BWC** | **IF&IHC** | **WB** | **Nissl**  **Staining** | **Golgi**  **Staining** | **Death** | **Excluded** | **Subtotal** | **Mortality** |
| --- | --- | --- | --- | --- | --- | --- | --- | --- | --- | --- | --- | --- |
| Experiment 1 |  | Sham | 5#+5 | 5* | 5 | 5 | 5 | 5 | 0 | 0 | 35 | 0 |
|  | 3d-4d | pMCAO  rMCAO | 8#+5  8#+5 | 7*  8* | 0  0 | 0  0 | 0  0 | 0  0 | 9+5*+4#  11+4*+6# | 1  3 | 42  45 | 42.9%(18/42)  46.7%(21/45) |
|  | 33d | pMCAO  rMCAO | 0  0 | 0  0 | 5  5 | 5  5 | 5  5 | 5  5 | 11  12 | 2  2 | 29  34 | 37.9%(11/29)  35.3%(12/34) |
| Experiment 2 | 10d | Naive | 0 | 0 | 5 | 5 | 0 | 0 | 0 | 0 | 10 | 0 |
|  |  | Sham | Shared with experiment 1 | | | | | | | | | |
|  |  | pMCAO  rMCAO+PBS  rMCAO+CLPs | 0  0  0 | 0  0  0 | 5  5  5 | 5  5  5 | 0  0  0 | 0  0  0 | 6  5  6 | 1  0  1 | 17  15  17 | 35.3%(6/17)  33.3%(5/15)  35.3%(6/17) |
|  | 33d | pMCAO | Shared with experiment 1 | | | | | | | | | |
|  |  | rMCAO+PBS | 0 | 0 | 5 | 5 | 5 | 0 | 6 | 1 | 22 | 27.3%(6/22) |
|  |  | rMCAO+CLPs | 0 | 0 | 5 | 5 | 5 | 0 | 7 | 1 | 23 | 30.4%(7/23) |
| Total |  | | | | | | | | 92 | 12 | 289 | 31.8%(92/289) |
